# Clinical outcomes and relapse patterns in pediatric acute leukemia patients undergoing hematopoietic cell transplantation: a multicenter Brazilian experience

**DOI:** 10.3389/fped.2025.1573334

**Published:** 2025-03-20

**Authors:** Polliany Roberta Dorini Pelegrina, Rita de Cassia Barbosa Tavares, Adriana Mello Rodrigues, Gisele Loth, Samantha Nichele, Cilmara Kuwahara, Fernanda Moreira de Lara Benini, Carolina Martins de Almeida Peixoto, Juliana Bach, Joanna Trennepohl, Lara Maria Miranda de Gouvea, Rafaella Muratori, Adriana Koliski, Rebeca Toassa Gomes, Marcia Quiroga, Simone Pereira Lermontov, Valeria Gonçalves da Silva, Ana Paula de Azambuja, Margareth Kleina Feitosa, Alberto Cardoso Martins Lima, Carmem Bonfim

**Affiliations:** ^1^ Pequeno Príncipe College, Curitiba, Paraná, Brazil; ^2^ Hospital Pequeno Príncipe, Curitiba, Paraná, Brazil; ^3^ National Cancer Institute (INCA), Rio de Janeiro, Rio de Janeiro, Brazil; ^4^ Hospital de Clínicas—UFPR, Curitiba, Paraná, Brazil; ^5^ Bone Marrow Transplant Unit, Hospital Nossa Senhora das Graças, Curitiba, Paraná, Brazil

**Keywords:** pediatric, ALL, AML, BMT, relapse

## Abstract

**Background:**

Hematopoietic cell transplantation (HCT) represents a well-established therapeutic strategy for high-risk leukemia, though post-transplant relapse remains a significant challenge, particularly in resource-limited settings

**Procedure:**

In this retrospective study, we analyzed medical records of 310 pediatric patients (age < 18 years) who underwent HCT for acute leukemias at four Brazilian institutions between 2010 and 2019.

**Results:**

The cohort included patients with acute lymphoblastic leukemia (ALL; 74.2%) and acute myeloid leukemia (AML; 25.8%). The median age was 9.52 years (range: 0.25–17.97), with male predominance (68.3%). Total body irradiation (TBI)-based conditioning was utilized in 72.6% of cases, and bone marrow served as the predominant graft source (74.5%). Prior to transplantation, 46.5% of patients were in first complete remission. Post-transplant relapse occurred in 36.7% of patients at a median of 245 days (range: 38–2,505). With a median follow-up of 2,019 days (5.5 years), overall survival was 69.3% at one year, declining to 62.4% at two years. The cumulative incidence of relapse was 12.6%, 28.8%, and 33.4% at 100 days, one year, and two years post-HCT, respectively. Adjusted analysis revealed increased relapse risk in patients with mixed donor chimerism, positive minimal residual disease (MRD) status before HCT, and disease status beyond first complete remission (including CR2, advanced disease, and refractory disease).

**Conclusion(s):**

These findings underscore the elevated relapse risk associated with advanced disease status, positive pre-HCT MRD, and mixed donor chimerism post-transplant. Future interventions should prioritize improving diagnostic capabilities, expanding access to modern treatment protocols, and facilitating early referral to transplant centers, particularly for aggressive disease presentations.

## Introduction

Hematopoietic cell transplantation (HCT) has emerged as a well-established therapeutic modality for diverse malignant and nonmalignant disorders. Recent decades have witnessed remarkable improvements in post-HCT outcomes, primarily attributed to advances in supportive care, high-resolution HLA typing technologies, and the expansion of stem cell donor registries. Despite its proven efficacy in enhancing cure rates for high-risk leukemia patients, achieving sustained remission remains challenging, particularly for those who experience disease recurrence within the first year post-transplantation (1, 2).

In high-income nations, post-HCT relapse represents the predominant cause of treatment failure and mortality, with cumulative incidence rates reaching 20% and 35% for Acute Lymphoblastic Leukemia (ALL) and Acute Myeloid Leukemia (AML), respectively (3). The prognosis following post-HCT disease progression remains poor, as evidenced by a comprehensive study of 279 pediatric cases revealing a median survival of merely 149 days (4). These findings underscore the critical need for effective relapse prevention and management strategies in high-risk populations. Notably, data from the Brazilian registry, encompassing over 5,000 patients with malignant diseases, presents a contrasting perspective to the Center for International Blood and Marrow Transplant Research (CIBMTR) findings (5). In the Brazilian context, infections, rather than relapse or organ failure, emerge as the primary cause of mortality within the initial 100 days post-HCT, highlighting the importance of considering regional healthcare disparities when analyzing post-transplant outcomes (6).

Currently, there is no standard recommendation for managing post-HCT relapse (7). This challenge is particularly pronounced in developing nations, where resource constraints often preclude access to innovative therapeutic options readily available in high-income countries. Such interventions include novel targeted agents like blinatumomab and inotuzumab, as well as advanced cellular therapies such as CAR T-cells, which could potentially serve as bridge-to-transplant strategies (8, 9) Understanding the characteristics associated with post-HCT relapse in resource-limited settings and identifying shared healthcare challenges becomes crucial for improving outcomes to match those observed in more developed nations.

Our investigation evaluated outcomes of hematopoietic cell transplantation in pediatric acute leukemia patients in Brazil's resource-constrained setting, especially regarding relapse patterns and risk factors. By identifying key predictors of transplant outcomes, we aim to develop strategies to improve long-term survival in this population.

## Methods

We analyzed the medical records of patients who underwent HCT for acute leukemias at four Brazilian institutions (Hospital Pequeno Príncipe, Hospital de Clínicas da UFPR, Hospital Nossa Senhora das Graças e Instituto Nacional do Câncer), between 2010 and 2019. All patients were younger than 18 years-old, and the diagnosis of the malignancy was established at the referring institution. The remission status was determined based on the latest available bone marrow evaluation before HCT. Patients were in remission if they had normal marrow cellularity and fewer than <5% blasts in the bone marrow, while those with ≥5% marrow blasts were classified as being in relapse. Measurable residual disease (MRD) was defined as any level of leukemic marrow blasts detected by available technology, including cytogenetics, molecular analysis, or multiparameter flow cytometry. Disease phase was categorized according to the number of medullary remission or relapse events prior to hematopoietic cell transplantation (HCT). Advanced disease was defined as patients who had achieved third or subsequent complete remission (≥CR3). For full compatibility: we consider 10/10 bone marrow and peripheral blood donors compatible, and 6/6 for cord blood. Prior to HCT, patients were treated according to standard protocols at each respective institution. Post-HCT relapse was defined as any morphologic, cytogenetic, or flow cytometry evidence of disease, at any detectable level in the bone marrow or at extra-medullary sites. A second hematologic malignancy without evidence of the original leukemia was not counted as a relapse. Neutrophil recovery was defined as the first of 3 consecutive days with an absolute neutrophil count (ANC) ≥ 0.5 × 10^9^/L. Platelet recovery was defined by a count of at least 20 × 10^9^/L, unsupported by transfusions, for seven days. Graft failure (GF) was defined as the failure to achieve an ANC of 0.5 × 10^9^/L by day 28 and absence of donor-derived hematopoiesis (<5% donor cells). Complete donor chimerism was defined as the presence of over 95% donor-originated cells, and mixed chimerism as the detection of 5%–95% donor-derived cells. Acute and chronic graft vs. host disease (GvHD) were scored according to previous published criteria (10, 11). Cytomegalovirus (CMV) reactivation was diagnosed by viral PCR or antigenemia, depending on the available diagnostic methods at each institution. Patients who were at risk of CMV reactivation included those in whom either the patient or donor, or both, had tested positive for CMV IgG. Haploidentical transplantation was performed using the post-transplant cyclophosphamide platform.

### Statistical methods

The median follow-up time was calculated using the reverse Kaplan–Meier method. Estimates of neutrophil and platelet recovery and GF were calculated using cumulative incidence curves to accommodate competing risks. Death without hematological recovery was considered a competing risk for neutrophil and platelet recovery, whereas death with sustained engraftment was a competing risk for GF. Relapse and NRM, which are both competing risks, were evaluated by the Gray Test and Fine-Gray competing risk regression. The Gray test was used for univariable comparisons of cumulative incidence curves. The following covariates were considered for univariable analysis: age, gender, year of transplant, ABO incompatibility, type of leukemia, disease status, MRD pre HCT, donor age, donor type, stem cell source, conditioning, use of serotherapy (ATG or campath), gender mismatch, GVHD prophylaxis, CMV reactivation, and donor chimerism. The overall survival (OS) was calculated from the day of the transplant until the date of death or the last contact with the patient, and the disease-free survival (DFS) was calculated from the day of the transplant until the day of relapse or death in remission. The probabilities of disease-free survival (DFS) and overall survival (OS) were estimated using the Kaplan–Meier method and compared between groups using the log-rank test. Multivariable analyses for neutrophil and platelet recovery and GF were performed using Fine-Gray sub distribution hazard models, whereas multivariable analyses for DFS and OS were performed using Cox proportional hazards regression models. Covariates that reached a *P* value ≤ 0.1 in the univariable analyses were included in the initial multivariable models and were eliminated one at a time using the backward stepwise method. Only covariates that attained a *P* value ≤ 0.05 (Wald test) were held in the final adjusted models. Results are expressed as the hazard ratio (HR) or sub distribution hazard ratio (SHR) with a 95% confidence interval (95% CI). A 2-sided *P* value ≤ 0.05 was considered statistically significant. Statistical analysis was performed using EZR version 1.53 (Saitama Medical Centre, Jichi Medical University, Saitama, Japan (12).

## Results

Table 1 summarizes patients' and transplantation characteristics. Between January 2010 and December 2019, we analyzed 310 pediatric patients who underwent their first hematopoietic cell transplantation (HCT) for acute leukemia. Acute lymphoblastic leukemia (ALL) represented the primary indication (74.2%), followed by acute myeloid leukemia (AML; 25.8%). The study population had a median age of 9.52 years (range: 0.25–17.97), with male predominance (68.3%).

**Table 1:** Patients' and transplant characteristics.

Characteristic	Group	Overall (*n* = 310)
Year of HCT		2016 (2010, 2019)
HCT Center (%)	1	83 (26.8)
	2	88 (28.4)
	3	49 (15.8)
	4	90 (29.0)
Age		9.52 (0.25, 17.97)
Sex (%)	Male	212 (68.4)
	Female	98 (31.6)
Type of leukemia (%)	ALL	230 (74.2)
	AML	80 (25.8)
ALL (%)	B	162 (52.3)
	T	56 (18,1)
	not applicable^a^	12 (3.8)
Disease phase (%)	CR1	144 (46.5)
	CR2	106 (34.2)
	Advanced Disease	60 (19.4)
Measurable residual disease pre HCT (%)	Negative	215 (69.4)
	Positive	69 (22.3)
	Active disease or unavailable	26 (8.4)
ABO (%)	compatible	165 (53.6)
	incompatible	143 (46.4)
Patient CMV (%)	IgG-	66 (21.3)
	IgG+	244 (78.7)
Donor age		26.00 (0.00, 55.00)
Donor type (%)	Related donor	92 (29.7)
	Unrelated donor	155 (50.0)
	Haploidentical donor	63 (20.3)
Sex mismatch (%)	No	181 (58.4)
	Female donor to male recipient	76 (24.5)
	Male donor to female recipient	53 (17.1)
Stem Cell Source (%)	BM	231 (74.5)
	PB	49 (15.8)
	UCB	30 (9.7)
HLA Compatibility (%)	Compatible (MRD or MUD)	184 (59.4)
	with any incompatibility	126 (40.6)
Conditioning (%)	TBI based	225 (72.6)
	Busulfan based	85 (27.4)
Serotherapy (%)	No	192 (61.9)
	Yes	118 (38.1)
GVHD prophylaxis (%)	CSA or TK + MTX	217 (70.0)
	CSA + corticosteroid or CSA + MMF or other	30 (9.7)
	PTCy + CSA + MMF	63 (20.3)
TNC UCB (×10.7/kg)		5.44 (3.50, 14.80)
TNC (×10.8/kg)		4.61 (0.28, 36.00)

MMF, mycophenolate mofetil; CSA, cyclosporine; MTX, methotrexate; TK, tacrolimus; BM, bone marrow, PB, peripheral blood, PTCy-post cyclophosphamide, MRD, matched related donor; MUD, matched unrelated donor; UCB, umbilical cord blood; TBI, total body irradiation; CR1, first complete remission; CR2, second complete remission; ALL, acute lymphoblastic leukemia; AML, acute myeloid leukemia; haplo, haploidentical; TNC, total nucleated cell.

^a^ MPAL or lineage switch.

Alternative donors were utilized in the majority of transplants (70.3%), with HLA compatibility achieved in 59.4% of cases. Bone marrow served as the predominant stem cell source (74.5%). Most recipients (94.8%; *n* = 294) were at risk for cytomegalovirus reactivation. The conditioning regimen consisted primarily of total body irradiation-based protocols (72.6%, TBI doses 1,200–1,440 rads), while busulfan-based regimens (doses 13–23 mg/kg) were used in the remaining patients (27.4%). Patients under 2 years of age (infant leukemia) received a modified conditioning regimen consisting of busulfan, fludarabine, and thiotepa, avoiding total body irradiation due to age-specific developmental considerations. Graft-vs.-host disease prophylaxis typically included a calcineurin inhibitor (cyclosporine or tacrolimus) combined with methotrexate (70%).

Regarding transplantation status, 46.5% of patients underwent the procedure while in first complete remission (CR1), and 69.4% had negative measurable residual disease analysis prior to HCT. Transplantation in CR1 was conducted in accordance with institutional protocols. Indications for CR1 transplantation were based on established high-risk features, including primary induction failure and adverse cytogenetic characteristics. Post-transplant relapse occurred in 36.7% of patients at a median of 245 days (range: 38–2,505). With a median follow-up of 2,019 days (5.5 years; 95% CI: 1,836–2,352), this cohort represents substantial longitudinal observation.

## Relapse

The cumulative incidence of relapse was 12.6% (95% CI: 9.2–16.5) at 100 days, increasing to 28.8% (95% CI: 23.8–33.9) at one year and 33.4% (95% CI: 28.2–38.7) at two years post-HCT. Time to relapse differed between acute lymphoblastic leukemia (ALL) and acute myeloid leukemia (AML) patients, with median times of 167 days (range: 22–2,504) and 100 days (range: 16–1,428), respectively.

### Risk factors for relapse

**Figure 1:**
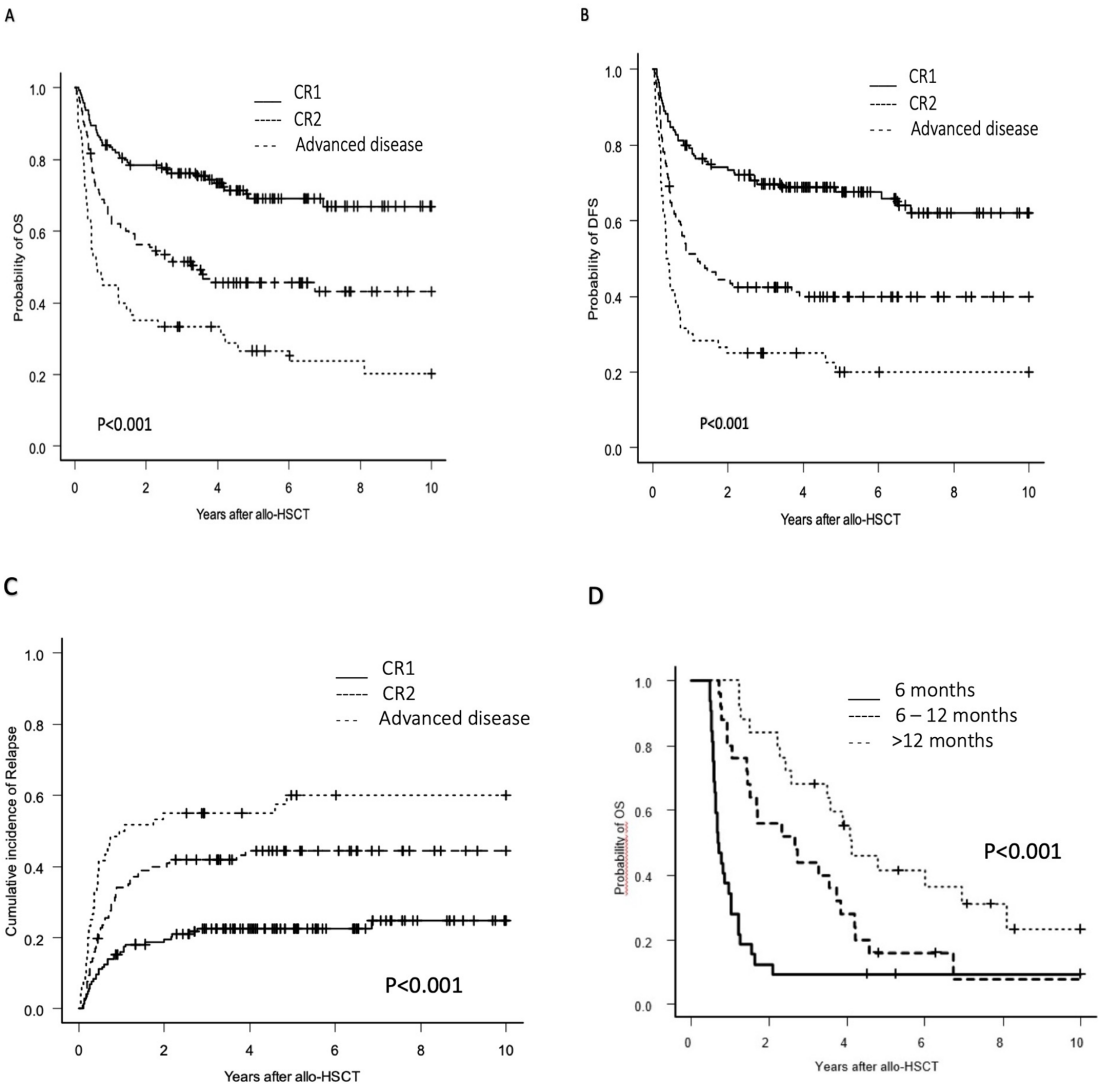
Overall survival, disease-free survival after HCT for pediatric patients with acute leukemias and incidence of relapse after HCT according to the disease phase and overall survival according to and time to relapse after transplant. **(A)** Probability of OS by disease phase, **(B)** Probability of DFS by disease phase, **(C)** Incidence of relapse by disease phase, **(D)** Probability of OS by time to relapse.

Univariate analysis (UVA) identified several factors associated with increased relapse risk: acute myeloid leukemia diagnosis, disease beyond first complete remission, positive minimal residual disease pre-HCT, and mixed donor chimerism at day +30 (Table 2). In multivariate analysis (MVA), mixed donor chimerism (HR 2.86, *p* = 0.047) and positive pre-HCT minimal residual disease (HR 2.26, *p* = 0.003) emerged as independent risk factors. Disease status beyond first complete remission showed a progressive increase in relapse risk, with hazard ratios of 2.39 (*p* = 0.0013) for second complete remission, 2.34 (*p* = 0.0026) for advanced disease, and 3.11 (*p* = 0.0006) for refractory disease (Table 3, Figure 1C).

**Table 2:** 1-year overall survival, disease-free survival, and relapse—univariate analysis.

Characteristics	Group	*n*	1-year OS	*p* value
Donor chimerism D + 30	Full donor chimerism	252	0.765 (0.708–0.813)	<0.01
	Mixed	8	0.375 (0.087–0.674)	
	Absent	13	0.385 (0.141–0.628)	
	Missing	37	0.378 (0.226–0.530)	
Disease phase	CR1	144	0.833 (0.762–0.885)	<0.01
	CR2	106	0.640 (0.540–0.723)	
	Advanced Disease	60	0.450 (0.322–0.570)	
MRD pre HCT	Negative	215	0.739 (0.674–0.792)	<0.01
	Positive	69	0.652 (0.527–0.752)	
	Active disease or unavailable	26	0.423 (0.235–0.600)	
Source	BM	231	0.731 (0.668–0.783)	0.015
	PB	49	0.653 (0.503–0.768)	
	CB	30	0.467 (0.284–0.630)	
Characteristics	Group	*n*	1-year DFS	*p* value
Disease phase	CR1	144	0.792 (0.716–0.849)	<0.001
	CR2	105	0.512 (0.412–0.603)	
	Advanced Disease	60	0.300 (0.190–0.418)	
MRD pre HCT	Negative	214	0.672 (0.605–0.731)	<0.001
	Positive	69	0.478 (0.357–0.590)	
	Active disease or unavailable	26	0.346 (0.175–0.525)	
Source	BM	230	0.629 (0.563–0.688)	0.041
	PBSC	49	0.592 (0.442–0.714)	
	UCB	30	0.400 (0.228–0.567)	
GVHD prophylaxis	CSA or TK + MTX	216	0.638 (0.570–0.698)	0.027
	CSA + corticosteroid or CSA + MMF or other	30	0.400 (0.228–0.567)	
	PTCy + CSA + MMF	63	0.571 (0.440–0.683)	
Donor chimerism D + 30	Full donor chimerism	251	0.661 (0.598–0.716)	<0.001
	Mixed chimerism	8	0.375 (0.087–0.674)	
	Absent chimerism	13	0.231 (0.056–0.475)	
	Missing chimerism	37	0.378 (0.226–0.530)	
Characteristics	Group	*N*	1-year Relapse	*p* value
Donor chimerism D + 30	Complete donor chimerism	252	0.251 (0.199–0.306)	0.037
	Mixed	8	0.625 (0.175–0.881)	
	Absent	13	0.308 (0.081–0.575)	
	Missing	37	0.459 (0.292–0.612)	
Disease phase	CR1	144	0.160 (0.105–0.225)	<0.001
	CR2	106	0.342 (0.252–0.433)	
	Advanced Disease	60	0.500 (0.366–0.620)	
MRD pre HCT	Negative	215	0.215 (0.162–0.272)	<0.001
	Positive	69	0.391 (0.276–0.505)	
	Active Disease Or Unavailable	26	0.615 (0.395–0.776)	

CR1, first complete remission; CR2, second complete remission; BM, bone marrow; PB, peripheral blood; UCB, umbilical cord blood; PTCy, post transplant cyclophosphamide; UCB, umbilical cord blood; MMF, mycophenolate mofetil; CSA, cyclosporine; MTX, methotrexate; TK, tacrolimus; ALL, acute lymphoblastic leukemia; AML, acute myeloid leukemia; haplo, haploidentical.

**Table 3:** Relapse—multivariate analyses.

Relapse	*n*	Hazard ratio	*p* value
Donor Chimerism D + 30	310		0.075
Full donor chimerism (reference)	252	1	
Absent chimerism	13	0.70 (0.25–1.93)	0.49
Missing chimerism	37	1.56 (0.92–2.66)	0.1
Mixed chimerism	8	2.86 (1.02–8.03)	0.047
Disease phase	310		<0.001
CR1 (reference)	144	1	
Advanced Disease	106	2.34 (1.35–4.06)	0.002
CR2	60	2.39 (1.53–3.73)	<0.001
Measurable residual disease pre HCT	310		<0.001
Negative	215	1	
Positive	69	2.26 (1.45–3.51)	<0.001
Active disease or unavailable	26	3.11 (1.62–5.96)	<0.001

CR1, first complete remission; CR2, second complete remission.

### Treatment and outcomes

Treatment strategies for post-transplant relapse included chemotherapy and immunosuppression withdrawal, frequently combined with donor lymphocyte infusions or second transplantation. In our cohort of 29 patients who underwent second transplantation, 19 received the procedure specifically for relapse management, with a median interval of one year between transplants. Only 3 of the 19 patients transplanted for relapse achieved sustained remission and survival.

## Additional analyses of the study population

### Overall survival

The estimated overall survival was 69.3% (95% CI: 63.8–74.1) at one year, 62.4% (95% CI: 56.7–67.5) at two years, and 52.7% (95% CI: 46.7–58.3) at five years post-HCT. UVA identified several favorable prognostic factors: first complete remission, negative minimal residual disease pre-HCT, ABO compatibility, bone marrow as stem cell source, and complete donor chimerism at day +30 (Table 2).

Due to violation of the proportional hazard assumption in the MVA, we performed a time-stratified analysis using a 365-day cutoff based on Schoenfeld residual assessment. In the early period (≤365 days), stem cell source emerged as the primary predictor of survival, with both peripheral blood (HR 2.23) and umbilical cord blood (HR 2.26) associated with increased mortality compared to bone marrow. Beyond 365 days, disease-related factors became predominant predictors of survival: second complete remission (HR 1.97), advanced disease (HR 3.18), and positive minimal residual disease pre-HCT (HR 3.12) were significantly associated with inferior outcomes (Table 4, Figures 1A,D).

**Table 4:** Overall survival before and after 365 days—multivariate analysis.

OS	<365 days		>365 days	
	Hazard ratio	*p* value	Hazard ratio	*p* value
Donor Chimerism D + 30		0.381		0.286
Full donor chimerism (reference)	1		1	
Mixed	1.49 (0.58–3.87)	0.41	3.17 (0.40–25.31)	0.28
Absent	1.69 (0.71–3.98)	0.23	2.45 (0.49–12.15)	0.27
Missing	1.45 (0.86–2.43)	0.16	0.30 (0.04–2.24)	0.24
Disease phase		0.315		0.007
CR1 (reference)	1		1	
CR2	1.16 (0.65–2.06)	0.61	1.97 (1.04–3.73)	0.037
Advanced Disease	1.58 (0.85–2.93)	0.14	3.18 (1.48–6.86)	<0.001
MRD pre HCT		0.372		<0.001
Negative			1	
Positive	1.41 (0.83–2.38)	0.2	3.12 (1.75–5.56)	<0.001
Active disease or unavailable	1.41 (0.71–2.77)	0.32	0.92 (0.22–3.25)	0.9
Stem cell source		<0.001		0.320
Bone marrow (reference)	1		1	
Peripheral blood	2.50 (1.45–4.32)	<0.001	1.00 (0.45–2.23)	1
Umbilical cord blood	2.98 (1.66–5.45)	<0.001	0.36 (0.09–1.37)	0.13

CR1, first complete remission; CR2, second complete remission; MRD, measurable residual disease.

### Rejection

The cumulative incidence of graft failure remained stable at 4.8% (95% CI: 2.8%–7.6%) from 100 days through one year post-transplantation. Although initial analysis suggested higher rejection rates in patients with busulfan-based conditioning, umbilical cord blood transplantation, and acute myeloid leukemia diagnosis, MVA identified only two independent risk factors: busulfan-based conditioning (HR 4.64, *p* = 0.005) and umbilical cord blood as stem cell source (HR 13.47, *p* = 0.000005).

### Disease-free survival (DFS)

Disease-free survival demonstrated a gradual decline over time, with rates of 60.1% (95% CI: 54.4–65.3) at one year, 54.2% (95% CI: 48.4–59.5) at two years, and 48.8% (95% CI: 42.9–54.4) at five years post-transplantation. UVA identified several favorable prognostic factors (Table 2): first complete remission status (46% of patients), negative minimal residual disease pre-HCT (69.3% of patients), bone marrow as cell source (74.4% of patients), and complete donor chimerism (81.2% of patients). In MVA, mixed donor chimerism emerged as a significant risk factor for inferior disease-free survival (HR 2.73, *p* = 0.019) compared to complete donor chimerism. Additionally, disease status beyond first complete remission significantly impacted outcomes, with both second complete remission (HR 2.52, *p* < 0.0001) and advanced disease (HR 3.58, *p* < 0.0001) associated with lower disease-free survival rates (Figure 1B).

### Acute GVHD (II-IV)

The cumulative incidence of acute graft-vs.-host disease (aGVHD) at day +100 was 35.0% (95% CI: 29.7%–40.3%) for grades II-IV and 14.3% (95% CI: 10.4%–18.9%) for grades III-IV. MVA identified unrelated donor transplantation as a significant risk factor, with 50% of these patients developing aGVHD (*p* = 0.0061).

### Chronic GVHD

The 2-year incidence of chronic GVHD was 23.0% (CI: 18,4%–27,8%), with severe cases accounting for only 5.4% (CI: 3.1%–8.7%). In the MVA, only age >10 years when compared to age <10 years, was a risk factor (47% of patients p 0.00007) for chronic GvHD.

## Mortality analysis

The study recorded 145 deaths at a median of 105 days post-HCT (range: 16–1,778). Relapse constituted the predominant cause of mortality (*n* = 102). Among the 43 patients who died in remission, infection was the primary cause in 90.6% of cases, with 11 of these patients having concurrent chronic GVHD. Temporal analysis of mortality patterns revealed distinct trends: infection dominated early deaths (58% within first 100 days), while relapse emerged as the leading cause thereafter (65% beyond day 100).

## Non-relapse mortality

The cumulative incidence of non-relapse mortality (NRM) showed a progressive increase from 6.8% (95% CI: 4.3–9.9) at 100 days to 11.9% (95% CI: 8.6–15.8) at one year, reaching 13.0% (95% CI: 9.8–17.3) at two years post-transplantation. UVA identified primary graft failure at day +30 (0.05% of patients, *p* = 0.003), haploidentical donors (20% of patients, *p* = 0.01), and umbilical cord blood as stem cell source (10% of patients, *p* = 0.001) as risk factors for increased NRM. MVA confirmed the independent impact of donor type and stem cell source on NRM: haploidentical donors (HR 4.00, *p* = 0.015), unrelated donors (HR 2.91, *p* = 0.026), and umbilical cord blood grafts (HR 3.5, *p* = 0.0012) were associated with significantly higher NRM rates.

## Discussion

Post-hematopoietic cell transplantation (HCT) relapse remains a significant challenge in pediatric acute leukemia treatment. Our study analyzed relapse rates and outcomes in a substantial cohort of pediatric patients, with particular emphasis on identifying critical factors that influence post-transplant success in resource-constrained settings. While post-HCT relapse is a global concern, data from Latin America and similar regions remain limited, creating a significant knowledge gap in understanding region-specific challenges and outcomes.

The relevance of this research is underscored by the high prevalence of acute leukemia in the pediatric population, with Brazil alone expecting approximately 11,000 new cases between 2023 and 2025. Understanding the patterns of post-HCT relapse and identifying both risk and protective factors is crucial for developing practical, resource-appropriate strategies for relapse prevention and management. This knowledge becomes particularly valuable for healthcare systems facing similar resource constraints, where optimizing available interventions can significantly impact patient outcomes (13).

In pediatric leukemia, post-HCT relapse rates demonstrate a strong correlation with disease status at the time of transplantation. While patients transplanted during first or second remission typically show more favorable outcomes, with reported rates between 23% and 30%, those undergoing transplantation with active disease face significantly poorer prognosis. Our findings align with these published data, underscoring the critical importance of achieving disease control prior to HCT (1, 2, 14–16).

The predictive value of minimal residual disease (MRD) for post-transplant relapse is well-documented (14, 17–19). Our findings reinforce the fundamental importance of achieving disease control before proceeding with HCT and highlight the need for standardized MRD assessment protocols (14, 20). A particular challenge emerges in resource-constrained healthcare systems where access to novel therapeutic agents that effectively achieve MRD negativity, such as blinatumomab and inotuzumab ozogamicin, remains restricted. In Brazil's public healthcare system (Sistema Único de Saúde, SUS), these agents are not currently available, and access is limited to patients with private health insurance coverage, who represent a minority of the Brazilian population. This disparity in access to novel therapies potentially compromises pre-transplant disease control for the majority of patients who rely on the public healthcare system (17).

Early withdrawal of immunosuppression (EWI) and donor lymphocyte infusions (DLI) were implemented as preventive strategies to enhance the graft-vs.-leukemia effect. While EWI was utilized in 58 patients, the concurrent use of multiple interventions and the retrospective nature of our study limited our ability to assess the individual effectiveness of these approaches. Although these strategies theoretically benefit patients with minimal disease burden, the optimal timing, dosing, and patient selection criteria remain challenging to establish. Furthermore, the potential benefits must be balanced against the risk of graft-vs.-host disease (4, 21).

Multiple studies have demonstrated varying outcomes with second hematopoietic cell transplantation as a therapeutic strategy for post-transplant relapse. While some reports show encouraging results, our experience aligns with retrospective studies showing more modest outcomes. The efficacy of this approach depends heavily on factors such as disease status at second transplant, time from first transplant to relapse, and donor availability, emphasizing the importance of careful patient selection (1, 22–24).

Our analysis demonstrates favorable post-transplant overall survival outcomes, aligned with contemporary literature. These results are comparable to those reported by Crotta, who observed rates of 77% and 60% at one and five years, respectively, and to Hoel's excellent outcomes for patients transplanted in complete remission (2, 7). Our findings also indicate significant progress in Brazilian transplant care when compared to the 45% three-year overall survival previously documented by Morando in 2010, likely reflecting improvements in supportive care and patient selection strategies (13) Disease status at transplantation and stem cell source emerged as critical determinants of outcomes. Our observation regarding the superiority of bone marrow transplantation, particularly within the first year post-transplant, corroborates Keesler's findings of improved outcomes with bone marrow grafts after six months post-procedure (25).

Disease-free survival analysis in our cohort identified mixed chimerism and primary graft failure as significant independent risk factors for inferior outcomes. Disease characteristics, including advanced disease status, second complete remission, and positive minimal residual disease pre-transplantation, demonstrated substantial impact on survival and relapse risk. These findings regarding disease-free survival align with previously published data in comparable populations, reinforcing the critical importance of disease control and successful engraftment in determining transplant outcomes (26, 27).

Non-relapse mortality in our cohort demonstrated favorable outcomes when compared to Wang and colleagues' findings (27). Our analysis identified donor type and stem cell source as significant determinants of transplant-related mortality. Specifically, haploidentical and unrelated donor transplants, as well as cord blood as a stem cell source, were associated with increased mortality risk. The higher mortality observed with cord blood transplantation may be attributed to an increased incidence of early rejection, potentially leading to greater susceptibility to infectious complications.

Our study's limitations stem from both methodological aspects and the complex healthcare landscape in Brazil. The country's continental dimensions and heterogeneous healthcare infrastructure create significant variability across leukemia treatment centers regarding diagnostic capabilities, treatment protocols, and timing of transplant referrals. This heterogeneity extends to transplant centers, where treatment options vary considerably between public and private healthcare systems, potentially influencing both pre- and post-transplantation care.

Additional limitations include the retrospective nature of our analysis, which inherently introduces potential biases in data collection and interpretation. The long study period may have encompassed evolution in supportive care practices and treatment strategies, potentially affecting outcomes across different time points. Furthermore, the assessment of minimal residual disease lacked standardization across centers, both in methodology and timing, which may impact the interpretation of this crucial prognostic factor. The evaluation of specific interventions, such as DLI and EWI, was challenging due to the variability in timing and dosing, as well as the concurrent use of multiple strategies. Additionally, our study's single-country focus, while providing valuable regional data, may limit the generalizability of findings to other healthcare settings with different resources and population characteristics.

Our study corroborates established risk factors for post-transplant relapse in pediatric acute leukemia while providing crucial data from the Brazilian healthcare context. Despite identifying previously known risk factors—advanced disease status, positive minimal residual disease pre-transplantation, and mixed donor chimerism—this analysis fills a significant knowledge gap regarding transplant outcomes in Latin American pediatric populations, where published data remains scarce. This regional perspective is particularly valuable given the unique challenges faced by emerging healthcare systems, including variable access to novel therapies and diagnostic tools.

The challenge of post-transplant relapse in resource-constrained settings highlights the importance of expanding access to novel therapies. While CAR-T cell therapy has emerged as a promising intervention for B-cell ALL relapse, its availability remains largely limited to the private healthcare sector in Brazil. Given the poor outcomes observed with second transplantation, developing academic CAR-T cell programs within the public healthcare system becomes imperative. Additionally, broader access to pre-transplant novel agents such as blinatumomab and inotuzumab could significantly improve disease control before transplantation.

Early infection-related mortality also warrants attention, particularly given the limited access to advanced antiviral medications like foscarnet and cidofovir in the public healthcare system. However, the primary focus remains on relapse prevention through improved early disease detection, timely transplant referral, and enhanced access to modern therapeutic protocols. These strategies, combined with standardized monitoring of minimal residual disease and chimerism, offer the most promising path toward improving long-term survival in pediatric acute leukemia patients undergoing transplantation.

Understanding transplant outcomes in our specific healthcare environment provides valuable insights for other regions facing similar resource constraints. This knowledge can guide the development of locally adapted strategies for relapse prevention and management, ultimately contributing to improved pediatric transplant outcomes across Latin America and other emerging healthcare systems.

## Data Availability

The raw data supporting the conclusions of this article will be made available by the authors, without undue reservation.
